# Immune Dysregulation, Polyendocrinopathy, Enteropathy, X-Linked Syndrome Associated With a Novel Mutation of *FOXP3* Gene

**DOI:** 10.3389/fped.2019.00020

**Published:** 2019-02-05

**Authors:** Charalampos Agakidis, Eleni Agakidou, Kosmas Sarafidis, Ioannis Papoulidis, Ioannis Xinias, Evangelia Farmaki

**Affiliations:** ^1^Pediatric Gastroenterology Section, First Department of Pediatrics, Ippokration General Hospital, Aristotle University of Thessaloniki, Thessaloniki, Greece; ^2^First Department of Neonatology, Ippokration General Hospital, Aristotle University of Thessaloniki, Thessaloniki, Greece; ^3^Access to Genome P.C., Clinical Genetics Laboratory, Thessaloniki, Greece; ^4^Pediatric Gastroenterology Section, Third Department of Pediatrics, Ippokration General Hospital, Aristotle University of Thessaloniki, Thessaloniki, Greece; ^5^Immunology Laboratory, First Department of Pediatrics, Ippokration General Hospital, Aristotle University of Thessaloniki, Thessaloniki, Greece

**Keywords:** autoimmune hepatitis, eczema, eosinophilia, hydrops fetalis, IgE, miscarriage, Treg cells, x-linked disorders

## Abstract

The immune dysregulation, polyendocrinopathy, enteropathy, X-linked (IPEX) syndrome is a rare, x-linked, recessive disorder characterized by dysfunction of the T regulatory (Treg) lymphocytes leading to autoimmune diseases. Herein we report a male patient with IPEX syndrome who presented with severe diarrhea, eczema, and malabsorption leading to failure to thrive and necessitating total parenteral nutrition, as well as with liver dysfunction. Laboratory investigation showed elevated liver enzymes that declined following treatment with glucocorticosteroids and immunosuppressive drugs, marked eosinophilia, increased total IgE, and decreased Treg cells. DNA analysis revealed that the patient himself was hemizygous and his mother heterozygous for the exon 10, c.1015C>T (p.Pro339Ser) mutation of the *FOXP3* gene, which has not been previously reported. The current case indicates that mutations resulting in substitution of a certain amino-acid (i.e., proline 339) by different amino-acids are manifested with different IPEX phenotypes.

## Introduction

The immune dysregulation, polyendocrinopathy, enteropathy, X-linked (IPEX) syndrome is a rare disorder characterized by dysfunction of the regulatory T_H_1 lymphocytes (Treg) that causes immune dysregulation leading to autoimmune diseases. It is an x-linked recessive disorder caused by mutations of the *forkhead box protein 3 (FOXP3)* gene that is located in the short arm of the x chromosome (Xp11.23). The *FOXP3* gene regulates production and function of the FOXP3 protein, which plays a key role in the Treg cell development and function (1–3). The disease mainly affects the intestine, the endocrine segment of the pancreas and skin, whereas blood cells, thyroid gland, kidneys, liver, and lungs may also be affected. The patients commonly present with early onset intractable diarrhea within the first days or months of life, refractory to dietary manipulation. Early onset diabetes type 1 and eczema may follow or precede the intestinal symptoms. Nevertheless, late onset cases with atypical presentation have also been reported (4).

At least 70 mutations of the *FOXP3* gene have been reported so far, accounting for a variety of disease phenotypes (5). Herein we report a male patient with IPEX syndrome who was hemizygous for the exon 10, c.1015C>T (p.Pro339Ser) mutation, a novel mutation of the *FOXP3* gene.

## Case Presentation

A male newborn, first live-born child of non-consanguineous, apparently healthy parents, was born at 37 weeks of gestation by spontaneous vaginal delivery. Maternal obstetric history revealed a miscarriage in the 11th week of gestation and an intrauterine death of a male fetus in the 30th gestational week due to hydrops fetalis. The family history for early infantile deaths and liver disease was unremarkable while there was a paternal family history of colon polyps and multiple food allergies. The current pregnancy was complicated by gestational diabetes managed with dietary counseling. Fetal ultrasound at 22 weeks of gestation was normal. At birth, meconium stained amniotic fluid was observed while the newborn developed respiratory distress and was admitted to the neonatal unit. The newborn had a birth weight of 2,800 g (10–50th centile), length 48 cm (50–90th centile), and head circumference of 35 cm (90th centile). The respiratory distress was treated with supplementary oxygen for 12 h. The newborn was discharged home on formula feeds, on the fifth day of life.

At 22 days of life, he was readmitted in neonatal intensive care unit for recurrent emesis and loose/watery stools. Clinical examination and blood biochemistry were unremarkable while a complete blood count revealed increased eosinophil number (6,000/μL). Abdominal ultrasound and upper gastrointestinal contrast study were normal. Brain magnetic resonance image performed as part of apneas work up was unremarkable. The patient was diagnosed with cow's milk protein allergy, and thereby was commenced on amino acid-based (elemental) infant formula resulting in improvement of gastrointestinal symptoms, and he was discharged home.

At the age of five and half months, he was admitted to a pediatric department for severe diarrhea (up to 10–15 watery bowel movements per day) during the preceding 2 months, and failure to thrive (weight <5th centile, length 10–25th centile, and head 90th centile). He underwent extensive investigation including colonoscopy which was unremarkable. The most notable laboratory findings were repeatedly elevated eosinophil blood counts ranging between 10 and 14% (absolute numbers 1,040–2,280 cells/μL) and liver enzymes (alanine aminotransferase 208 IU/mL and aspartate aminotransferase 337 IU/mL). Serum levels of CRP and erythrocyte sedimentation rate were normal. Endocrine test results (thyroid stimulating hormone, free triiodothyronine, free thyroxin, ACTH, cortisol, and 17-hydroxylproline) were normal while tests for viral infections (cytomegalovirus, hepatitis B, adenovirus, as well as Ebstein Bar, coxsackie, and rota viruses) were negative. Immunologic tests revealed a markedly elevated total IgE (6,650 IU/mL), normal immunophenotype (not including Treg cells), neutrophil phagocytosis, and intracellular bacteria killing as well as negative anti-nuclear autoantibodies. Stool analysis was unremarkable.

**Figure 1:**
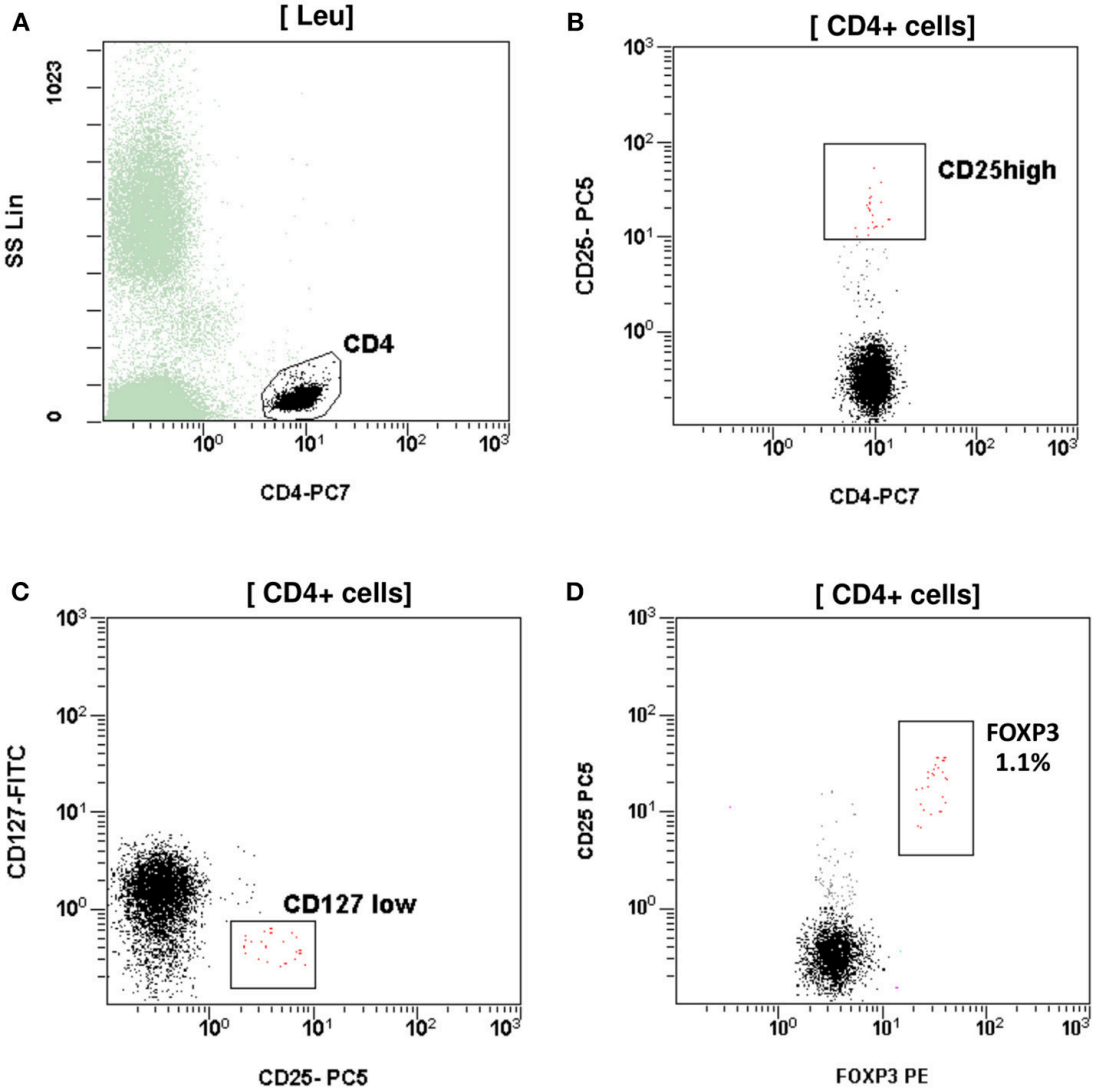
T regulatory cells (Tregs) assessed as CD4+CD25^high^CD127^low^FOXP3+. **(A)** CD4+ T cells gated by CD4 and side scatter (SS). **(B)** Treg CD4+CD25^high^. **(C)** Treg CD4+CD25+CD127 ^low^. **(D)** Treg CD4+CD25+FOXP3+. The percentage (1.1%) and absolute number (20 cells/mmL) of Treg was reduced compared to reference age values (4–8% and 63–690 cells/mmL respectively).

**Figure 2:**
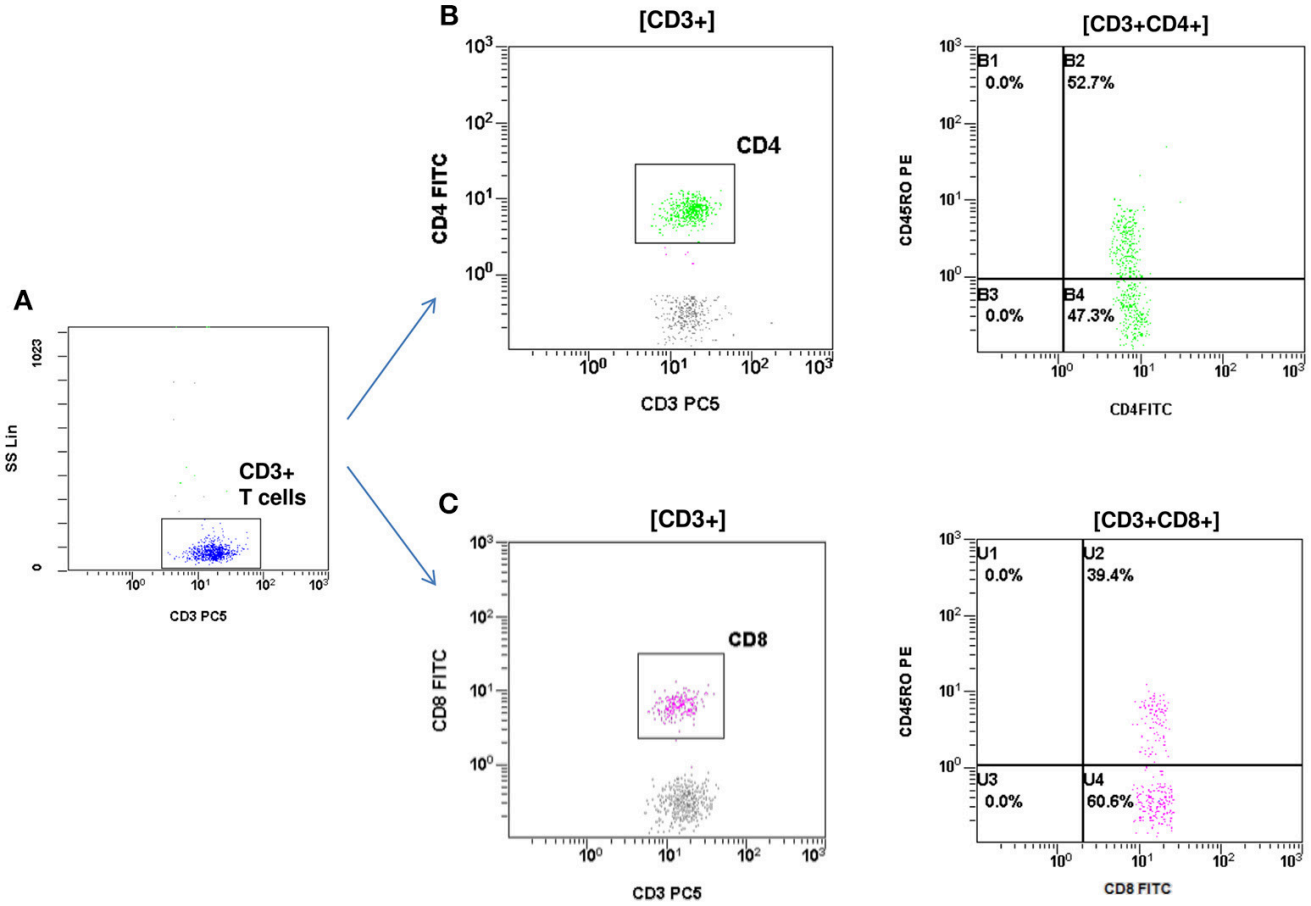
Flow cytometric assessment of memory T helper and memory T cytotoxic cells. **(A)** T cells were gated by CD3 and side scatter (SS). **(B)** T helper memory cells: CD3+CD4+CD45RO+. **(C)** T cytotoxic memory cells: CD3+CD8+CD45RO+.The percentage of memory T helper (52.7%) and memory T cytotoxic cells (39.4%) were elevated compared to reference age values (7–20 and 2–15% respectively).

At the age of 7 months, he had weight <3rd centile, 10–15 voluminous watery diarrheas daily, abdominal distension, enlarged liver (+2 cm), and marked anal excoriation. Laboratory tests showed iron deficiency anemia with low ferritin, elevated eosinophils, elevated aminotransferases (300–400 IU/L). Immunology investigation showed normal levels of IgG, IgM, C3, and C4, and markedly elevated total IgE (11,600 IU/mL). Immunophenotyping showed: (a) decreased percentage of FOXP3 positive Treg cells (CD4+CD25+CD127^low^FOXP3+) at 1% of total CD4+ cells (age reference values 4–8%) and an absolute number of 20 cells/mmL (age reference values 63–690 cells/mmL), (b) elevated percentages of memory T helper cells (CD4+CD45RO+) at 52.7% and memory T cytotoxic cells (CD8+CD45RO+) at 39.4% compared to age reference values (7–20 and 2–15%, respectively), and (c) decreased naïve T helper cells (CD4+CD45RA+) at 47.3% and naïve T cytotoxic cells (CD8+CD45RA+) at 60.6% compared to age reference values (64–93 and 70–93%, respectively) (Figures 1, 2). Immunophenotyping was otherwise normal. Tests for antinuclear, antiislet cell, antiendomysial, antithyroid peroxidase, anti-thyroglobulin, antiglutamic acid decarboxylase, antitissue-transglutaminase, and antienterocyte antibodies were negative, while the antireticulin antibodies were positive (1/160). Macroscopic findings of upper endoscopy and colonoscopy were normal, while pathology revealed non-specific inflammation of the stomach, duodenum, and colon, which was more prominent in the duodenum. The patient was given a provisional diagnosis of autoimmune enteropathy, possibly in the context of IPEX syndrome, and genetic testing was performed. Subsequently, he was started on methylprednisolone 1 mg/kg long term that resulted in clinical improvement, and total parenteral nutrition (TPN). Within a few days of immunosuppression, he had 3–4 bowel movements per day of normal consistency, without visible blood or mucous, and was gaining weight. TPN was discontinued after a couple of weeks. During the introduction of solid foods, which followed the phase of clinical stabilization, he experienced anaphylactic reactions to several food allergens, namely milk, beef, and wheat. He was tested positive for all these allergens (RAST class 5). He was also commenced on tacrolimus (target levels 10–15 ng/mL) in an attempt to wean him from methylprednisolone but this made little clinical impact as the patient deteriorated in every attempt to reduce methylprednisolone below 1 mg per kg.

**Figure 3:**
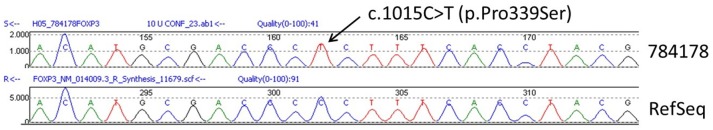
Confirmatory Sanger Sequencing of the FOXP3 c.1015C>T (p.Pro339Ser) mutation from the patient in relation to the reference sequence NM_014009.3 (RefSeq).

The sequence analysis of FOXP3 gene revealed that the patient was hemizygous for the exon 10, c.1015C>T (p.Pro339Ser) mutation (Figure 3), which is regarded as pathogenic although it has not been described previously in the literature. His mother was heterozygous for the same mutation. The infant was discharged home on methylprednisolone, tacrolimus, and elemental formula feedings, with intravenous catheter *in situ*. On discharge, he was in good condition with a body weight at the 3rd centile.

Two admissions followed in the next few months for catheter-related sepsis and hypoalbuminemia, which were treated appropriately. Moreover, skin lesions compatible with chronic eczema were apparent on extremities and genitalia.

At the age of 13 months, he was referred to a bone marrow transplantation center to be registered in a bone marrow transplantation awaiting list. A second DNA analysis by another laboratory confirmed the *FOXP3* mutation. In essence during this hospital stay, the patient was switched from tacrolimus to sirolimus (rapamycin) in an attempt to wean him off methylprednizolone. However, while on sirolimus, he developed sepsis and aphthous mouth ulcers, before switching back to methylprednisolone and tacrolimus. However, it is a fact that intestinal inflammation was not adequately controlled even with the combination of tacrolimus and methylprednisolone. Clinically, his weight remained static at 8 kg approximately (<3rd centile) and he experienced recurrent episodes of diarrhea (1,200 mls per day) while on elemental formula feeds via nasogastric tube and small amounts of fruits, vegetables, and meat. In addition, a new endoscopy and biopsies performed at the age of 21 months showed intense inflammatory infiltrations of neutrophils, lymphocytes, plasma cells, and eosinophils in the colon, stomach, and duodenum, where rare small crypt abscesses were also observed without any villous architecture distortion, as well as focal pyloric metaplasia in the terminal ileum. Soon afterward, his clinical status deteriorated necessitating hospitalization for stabilization and correction of hypoalbuminemia and metabolic acidosis. TPN was commenced once again, but he experienced two episodes of catheter-associated sepsis requiring intensive care. At the age of two and half years he was transferred abroad to a bone marrow transplantation center where he was subjected to bone marrow transplantation from a 100% compatible donor. Currently, 4 months post-transplantation, the patient is still hospitalized in the transplant center. We have confirmed that engraftment was successful, patient has remained clinically stable and free of any major complications, is gaining weight very satisfactory with no TPN and has been weaned off methylprednisolone.

## Discussion

The clinical, immunology, and histological features of the current patient are consistent with IPEX syndrome. DNA analysis revealed a novel mutation of the *FOXP3* gene resulting in substitution of proline 339 by serine. In the context of previously reported mutation of the *FOX3* gene leading to substitution of proline 339 by alanine in IPEX patients, the clinical, histological, and laboratory findings of the current patient could be related to the novel mutation of the *FOXP3* gene.

Studies of families with members affected by IPEX syndrome documented an association of hydrops fetalis and miscarriages with prenatal onset of autoimmunity resulting in fetal demise (6–9). In the current case, maternal obstetric history revealed a miscarriage at 11 weeks of gestation and an intrauterine death of a male fetus with hydrops fetalis on the 30th week of gestation. Although no investigation or pathology exam was performed on the lost fetuses, it is very likely that at least the hydropic fetus experienced IPEX syndrome.

Autoimmune enteropathy is the most common manifestation of IPEX syndrome, usually presenting within the first days or weeks of life with intractable watery diarrhea. In our patient, the episodes of diarrhea started around the 15th day of life, while being on elemental formula feedings, and they rapidly deteriorated causing severe failure to thrive by the fifth month of life. Vomiting, gastritis, and colitis (10–13) with non-specific gut mucosal infiltrations on histology examination have been previously described in IPEX patients while metaplastic lesions and abscesses have also been reported (1, 5). In agreement with previous reports, the upper endoscopy and colonoscopy findings before implementation of immunosuppressive (IS) therapy revealed non-specific inflammatory infiltration in the stomach and duodenum, which were compatible with IPEX syndrome. A follow-up endoscopy 1 year later, while on IS therapy, showed deterioration of the inflammatory infiltrations in the duodenum, while severe non-specific inflammation was found in the colon along with crypt abscesses and metaplasia lesions in the terminal ileum.

The skin manifestations found in the current case and previous IPEX patients include eczematiform dermatitis and other less common dermatitis (ichthiasiform and psoriasiform) (14). In addition, our patient exhibited urticarial rash during acute allergic reactions, usually associated with the introduction of a new food.

Autoimmune hepatitis associated with autoantibodies has been described in patients with IPEX syndrome. The current patient presented with increased serum levels of aminotransferases but normal gamma-glutamil transpeptidase and serum bilirubin at the age of 5 months. Hepatitis caused by known viruses, hepatotoxic medications, or TPN (liver dysfunction preceded TPN implementation) were ruled out (15). These findings combined with the decrease in liver enzymes following initiation of IS treatment support the autoimmune nature of the liver dysfunction.

Various autoimmune disorders including diabetes type 1 (5, 11–13, 16), thyroid dysfunction, blood cytopenias, interstitial pneumonitis potentially leading to acute respiratory distress syndrome (17), and renal disease (18–21) have been reported in IPEX patients. Our patient had no clinical and laboratory evidence of the above autoimmune disorders up to the age of two and half years, while the tests for a variety of autoantibodies were negative. Nevertheless, these complications could emerge over the course of the disease.

### Diagnosis

The clinical, biochemical, and gastrointestinal histology findings raised the suspicion of IPEX syndrome, which was reinforced by immunology tests. Immunophenotyping revealed decreased percentage and absolute numbers of Treg cells positive for the FOXP3 protein (CD4+CD25+CD127^low^FOXP3+). Treg cells have inhibitory immune properties thereby being important for maintaining self-tolerance and immune homeostasis (22, 23). The low number of Treg cells found in our patient partly explains the autoimmune disorders observed. Moreover, Treg cells isolated from our patient could be dysfunctional, as has been demonstrated by previous authors (5, 10, 13, 24).

To confirm diagnosis of IPEX syndrome, it is necessary to identify mutations of *FOXP3* gene. Sequencing of the *FOXP3* gene (chromosome locus Xp11.23) has revealed that the patient is hemizygous for exon 10, c.1015C>T (p.Pro339Ser) mutation (reference sequence: NM_0140009.3). In addition, the DNA analysis of maternal blood revealed that she is heterozygous for the same mutation of the *FOXP3* gene. These results have been confirmed by two different genetic labs. The current mutation has not been found in other IPEX patients or in any population databases (dbSNP, gnomAD, ESP, ExaC, etc). The mutation affects a proline molecule (proline 339) that is located in a gamma-turn between Arg337 and helix 1 at the amino-terminal part of the Forkhead DNA-binding domain (16). Proline 339 is 100% conserved across all species (25). Therefore, it is likely that changing this to another amino acid would have consequences. In fact, three IPEX patients have been reported with a mutation concerning substitution of proline 339 by alanine (c.1015C>G [p.Pro339Ala]), but not serine as in our patient (11, 13, 16). It has been suggested that substitution of proline 339 by alanine potentially modifies the way in which Arg337 and/or helix 1 is arranged in relation to the DNA thereby affecting the DNA binding affinity (16). Moes and others reported that an IPEX patient with exon 10, c.1015C>G mutation showed cytosolic FOXP3 expression without nuclear localization, as observed in other IPEX patients with missense mutation in the middle part of exon 10 and in healthy controls (13). These findings indicate that the N and C-terminal parts of the Forkhead domain are important for the nuclear import of FOXP3 (21). Moreover, proline residues are frequently associated with creating bends in proteins to allow protein folding. Based on these data, it is likely that the current mutation affects the structure of the Forkhead DNA-binding domain of the protein and should be regarded as pathogenic. Although the way in which substitution of proline 339 by serine affects FOXP3 protein function has not been explored, it is possible that this mutation is associated with the clinical and laboratory features of IPEX syndrome of the current patient.

All the IPEX patients previously reported with different mutations affecting the same proline molecule, that is substitution of proline 339 by alanine, presented with early onset of diabetes type 1 and enteritis, with villous atrophy in one patient that responded to gluten-free diet, and growth failure (11, 13, 16). Other manifestations included eczema in two patients (13, 16), autoimmune hemolytic anemia in two (11, 13), hypothyroidism in one (16), and autoimmune hepatitis in one patient (11). No patient experienced allergy, while all died within the first 7 months of life.

### Treatment and Outcome

In the management of IPEX patients, physicians of different expertise should be involved, since several systems may be affected. Depending on the type and severity of autoimmune enteritis, supportive treatment includes parenteral fluids and nutrition. Substitution therapy is needed in the case of protein losing enteropathy, endocrinopathies, and blood cytopenia. Our patient required albumin infusions during the second year of life and intermittent total or partial parenteral nutrition as well as iron and magnesium supplementation. One or more IS drugs may be required for treatment of IPEX patients. Drugs that are most frequently used include glucocorticosteroids, mainly methylprednizolone, and IS drugs, mainly calcineurin inhibitors (tacrolimus and cyclosporine), azathioprine, and rapamycin (sirolimus) (5, 11). Currently, bone marrow and allogeneic hematopoietic stem cell transplantation are considered as the only potentially curative therapies for the IPEX syndrome (26). In our patient, treatment with methylprednizolone quickly resolved diarrhea and liver dysfunction, and promoted weight gain. However, every attempt to taper methylprednizolone and replace it by IS drugs failed. Therefore, the patient was treated with methylprednizolone combined with one IS drug, which was either tacrolimus, or rapamycin, or azathioprine. Nevertheless, at the age of 20 months, histology of intestinal biopsies showed deterioration of the intestinal inflammation despite the intense IS treatment. For this reason, he was subjected to bone marrow transplantation.

The prognosis of IPEX syndrome is variable, albeit generally poor. An international multicenter retrospective study, which was conducted by the Primary Immune Deficiency Treatment Consortium (PIDTC) and the Inborn Errors Working Party (IEWP) of the European Society for Blood and Marrow Transplantation (EBMT), showed that the survival rate of IPEX patients is 65% after IS treatment and 73% after hemopoietic stem cell transplantation (1). The only significant predictor of survival was the number and severity of organ involvement. In the current patient, the syndrome had little or no effect on organs other than the gastrointestinal tract, which is associated with a more favorable outcome. However, the extensive involvement of both upper and lower gastrointestinal tract, combined with the lack of effectiveness of IS monotherapy, is indicative of an unfavorable prognosis, especially if the patient is not offered a bone marrow or hemopoietic stem cell transplantation.

## Concluding Remarks

Findings of the current patient suggest that the exon 10, c.1015C>T mutation of the *FOXP3* gene leading to substitution of proline339 by serine is associated with IPEX syndrome, mainly manifested with severe enteritis and failure to thrive. The novel *FOXP3* mutation described herein was not associated with early diabetes type I, which was a constant finding in previously reported IPEX patients with *FOXP3* gene mutation concerning substitution of the same molecule of proline339 by alanine instead of serine. The current case indicates that mutations resulting in substitution of a certain amino-acid (i.e., proline 339) of the FOXP3 protein by different amino-acids are manifested with different IPEX phenotypes.

## Parental Consent

Written parental consent for the publication has been obtained.

## Author Contributions

CA and EA conceptualized and wrote the article under the supervision of KS and EF. CA, EA, and IX performed gastrointestinal endoscopy and evaluation, treated and followed up the patient. EF performed and interpreted the immunological tests. IP performed and interpreted the genetic tests. KS and EF critically revised the manuscript.

### Conflict of Interest Statement

IP is employed by company “Access To Genome P.C., Clinical Genetics Laboratory.” The remaining authors declare that the research was conducted in the absence of any commercial or financial relationships that could be construed as a potential conflict of interest.

## Abbreviations

FOXP3: Forkhead Box Protein 3
IPEX: Immune dysregulation, Polyendocrinopathy, Enteropathy, X-linked
IS: Immunosuppression
TPN: Total Parenteral Nutrition
Treg: T regulatory cells.
